# Seasonal Evaluation of Antigenic Bacterial Infections Among Working Class in the Inner City of Houston

**DOI:** 10.1100/tsw.2004.135

**Published:** 2004-08-31

**Authors:** Ebere C. Anyanwu, Mohammed Morad, Andrew W. Campbell

**Affiliations:** ^1^ Neurosciences Research, Cahers Inc, 8787 Shenandoah Park Drive, Suite 122, Conroe, Houston, TX 77385, USA; ^2^ Clalit Health Services and Division of Community Health, Department of Family Medicine, Ben Gurion University, Beer-Sheva, Israel; ^3^ Medical Center for Immune and Toxic Disorders, 20510 Oakhurst Drive, Suite 200, Spring, TX, USA

**Keywords:** bacterial infections, Streptococcus pneumoniae, Haemophilus influenzae b, Streptococcus, Neisseria meningitides, screening methods, public health

## Abstract

This paper evaluates the monthly, quarterly, and seasonal variation of antigenic bacterial infections among the working class in the inner city of Houston using the Wellcogen Rapid Test methods. One of the aims was to demonstrate how this method could be used effectively in screening patients at risk and preventing the spread of antigenic bacteria such as *Streptococcus pneumoniae*, *Haemophilus influenzae* b, *Streptococcus* (Strep b), and *Neisseria meningitidis* (mainly group c and b). A total of 2,837 patients were screened for bacterial infections; 908 (32%) were male and 1,929 (68%) were female. The age range was between 2 and 70 years. Of the total group, 356 (12.5%) patients were positive; 203 (57%) were female while 153 (43%) were male (male/female ratio of 1:1.3). Medically underserved and immune suppressed populations are the most affected by these bacterial infections. Blacks are the most affected (48%) compared to Native Americans (1%), but children under 10 years of age have the highest incidence. This research showed, in addition, that the Wellcogen Rapid Tests are effective (356 cases identified) for a rapid screening of infectious bacteria. Explanation for these results was probably due to poor living conditions, poor hygiene, and viral immune suppression in adults and immature immune systems in neonates and children under 10 years of age.

## INTRODUCTION

Systematic virologic surveillance in Houston over the past 15 years has revealed that influenza virus infections have been epidemic during each respiratory disease season. The peak of the occurrence of acute respiratory illnesses that caused patients to seek medical care always coincided with the peak of influenza virus activity[1]. The peak number of deaths attributed to pneumonia or influenza followed the peak of influenza virus activity by about 2 weeks. This relationship was constant even though the time of the epidemic peak ranged from as early as the third week of December to as late as the second week of March[1]. Therefore, the methods used to predict baseline mortality that showed a seasonal rise in the absence of influenza activity may be inaccurate and could lead to underestimation of mortality associated with influenza virus infections[1].

One of the most difficult problems the public health authorities face every day is being able to identify the incidence of infectious disease at the symptomatic stages. A few years ago in the Midlands, U.K., some school children from the inner cities died as a result of meningococcal infection. If the initial symptoms of the meningococcal attack had been identified earlier and treatment started, these children might have survived. The seasonal outbreak of bacterial infection all over the world is published in many internationally public health-related journals. For example, *Streptococcus, Haemophilus influenzae* b, *S. pneumoniae*, and *Neisseria meningitidis* c and b dominate the bacterial infectious outbreaks in the U.S.[2] in a decreasing order of prevalence.

The seasonal variation is influenced by a number of prevailing factors. These include environmental conditions, standard of living, personal hygiene, and immune response. Several anecdotal accounts show that neonates and children under 10 years of age are most affected by these factors. Poor medical attention and an immature immune system expose the neonates and the children to infections during the time of birth. Adults who are affected the most are those that live under poor housing conditions and congested living facilities. In the light of these conditions, bacterial infections cannot be ruled out. *S. pneumoniae* are commonly referred to as “pneumococci” and were formerly classified in the genus *Diplococci*. The organisms produce infections in various organs and tissues, the most common and serious being lobes bronchopneumonia, involving part or whole lobes of the lungs. They attack both the very young and healthy adults. Of all bacterial pneumonia, 80% are caused by pneumococcus[2].

*H. influenzae* is an important cause of meningitis in children. It causes obstructive bronchitis in infants and children and has continued to be the frequent cause of a variety of serious infections in young children because of their poor immune response and increasing emergence of drug-resistant organisms. The most virulent is the *H. influenzae* type b, which causes 95% of the acute infections involving deep tissue invasion, including meningitis, primary segmental or lobar pneumonia, laryngotracheitis, epiglottitis, cellulites, and purulent arthritis[2]. *N. meningitidis* causes cerebrospinal meningitis, which often causes death a few hours after the first symptoms appear. Epidemics of meningococcal meningitis occur frequently under circumstances in which large numbers of individuals, especially from different geographical regions, are brought together under crowded and stressful conditions.

The aim of this research is to demonstrate the effectiveness of rapid tests in the screening and the prevention of the spread of these infectious bacteria among those who are medically underserved.

## METHODS

The hospital laboratory where this research was conducted served mostly the underprivileged and the medically underserved population. A total of 2,837 patients were screened for bacterial infection at a Houston, Texas general hospital for 12 months, from January to December 1991. The Wellcogen Rapid Latex Tests reagents (Code Nos.: ZL20, ZL21, ZL24)[3] were used. These rapid latex tests reagents are for use in the qualitative detection of antigens from Strep b, *H. influenzae* b, *S. pneumoniae* and *N. meningitidis* group b, and *Escherichia coli* K1, respectively.

The patients screened were classified according to age, sex, and ethnicity. Samples of body fluids (urine, serum, cerebrospinal fluid [CSF], and blood) from these patients were processed as described in the test and quality control procedures by Wellcome Diagnostics, a Division of the Wellcome Foundation Limited, Dartfort, England. The samples were analyzed, interpreted, and recorded as either positive, negative, or noninterpretable. Those negative and noninterpretable results were further cultured to exclude experimental error, using blood agar plate procedures. Monthly and quarterly data were then tabulated and analyzed as shown in the tables below.

## RESULTS

The monthly values of bacterial *H. influenzae*, *S. pneumoniae*, and *N. meningitides* antigen positives, using Latex Tests by Wellcome are presented in Tables 1–3 and in Fig. 1. Overall, the Wellcogen Rapid Test method was significantly effective in identifying positive cases of antigenic bacterial infections.

**TABLE 1**
**Monthly Values**

| Number | Month | Positive Cases |
|---|---|---|
| 1 | January | 38 |
| 2 | February | 36 |
| 3 | March | 37 |
| 4 | April | 37 |
| 5 | May | 34 |
| 6 | June | 21 |
| 7 | July | 17 |
| 8 | August | 16 |
| 9 | September | 18 |
| 10 | October | 38 |
| 11 | November | 21 |
| 12 | December | 43 |
| **Total** | | **356** |

Of the total number of patients screened (2,837), 908 (32%) were male and 1,929 (68%) were female. The age range was between 2 and 70 years. Of the total group, 356 (12.5%) patients were positive; 64 (18%) were white, 170 (48%) were black, 110 (31%) were Hispanic, 7 (2%) were Asian/Indian, while only 5 (1%) were Native American.

Of the positive group, 203 (57%) were female, while 153 (43%) were male (male/female ratio of 1:1.3). Children under the age of 10 were the most affected (179 cases). The least infected were between the age groups 61 through 70 years.

Streptococcal infection made up 46% of the total number of positives (146); 28% (98) was due to *H. influenzae* b, 17% (62) was due to *S. pneumoniae*, while 14% (50) was due to *N. meningitidis* group c (Table 2).

**TABLE 2**
**The Distribution of Positives By Ethnicity**

| Type of Bacteria | White, Not Hispanic | Black, Not Hispanic | Hispanic | Asian/Indian | Native American | Total |
|---|---|---|---|---|---|---|
| | No. (%) | No. (%) | No. (%) | No.(%) | No. (%) | No. (%) |
| Strep b | 25 (16) | 83 (56) | 37 (25) | 1 (0.7) | 1 (0.7) | 146 (41) |
| *H. influenzae* b | Nil 10 (10) | 49 (50) | 32 (33) | 5 (5) | 2 (2) | 98 (28) |
| *S. pneumoniae* | 15 (24) | 20 (28) | 23 (37) | 1 (2) | 3 (5) | 62 (17) |
| *N. meningitidis* | 14 (28) | 18 (36) | 18 (36) | 0 (0) | 0 (0) | 50 (14) |
| **TOTAL** | **64 (18)** | **170 (48)** | **110 (31)** | **7 (2)** | **5 (1)** | **356 (100)** |

**TABLE 3**
**Quarterly Distribution of Bacterial Positives by Age**

| | Quarter | | | | |
|---|---|---|---|---|---|
| Age (Years) | 1/(%) | 2/(%) | 3/(%) | 4/(%) | Total No./(%) |
| Under 10 | 60 (35) | 35 (20) | 29 (16) | 55 (31) | 179 (50) |
| 11–20 | 25 (37) | 12 (18) | 14 (21) | 17 (25) | 68 (19) |
| 21–30 | 14 (30) | 17 (36) | 2 (4) | 14 (30) | 47 (13) |
| 31–40 | 5 (21) | 9 (38) | 4 (17) | 6 (25) | 24 (7) |
| 41–50 | 4 (22) | 6 (33) | 2 (11) | 6 (33) | 18 (5) |
| 51–60 | 1 (7) | 10 (71) | 0 (0) | 3 (21) | 14 (4) |
| 61–70 | 2 (33) | 3 (50) | 0 (0) | 1 (17) | 6 (2) |
| **TOTAL** | **111 (31)** | **92 (26)** | **51 (14)** | **102 (29)** | **356 (100)** |

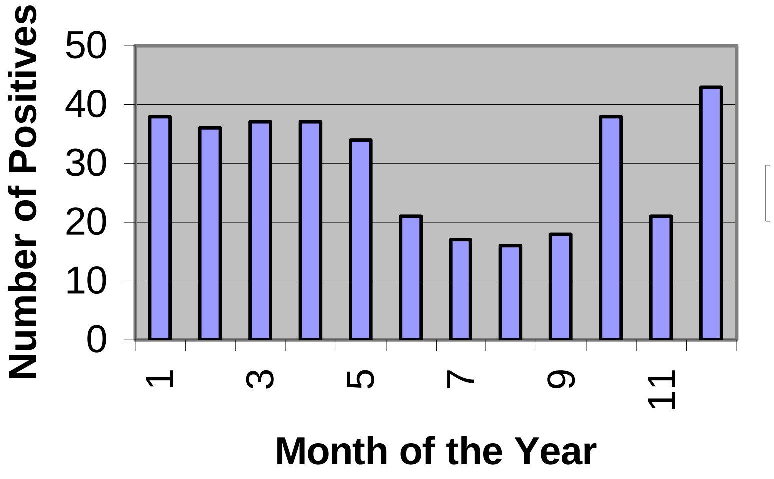

**FIGURE 1.** The monthly variation.

The monthly and quarterly presentations of the cases (Tables 1 and 2) showing the highest incidence among those under the age of 10 years was in the months of January, October, November, and December. The results revealed a peak period of infection for the age groups 11–20 years of age in the months of March and December, 21–30 in the months of February and May, 31–40 in the month of June only. The highest incidence among those aged 41–70 was between April and May. The mean was 29.67 and the standard deviation 10.08.

## DISCUSSION

Any bacterial infectious control efforts by the public health authorities must address principally three issues: (1) the population of the society that is at risk of infectious diseases, (2) the seasonal variation of such infections, and (3) which etiological agents are involved as the cause of the infections, for example, meningitis, pneumonia, bronchitis, or bronchopneumonia. Overcrowding is known to be a risk factor for meningococcal disease especially in children, where the risk increases with increasing household density[4], and this mode of living adversely affects preschool children and older adults. An association was shown to exist between meningococcal disease and influenza[5]. Several other factors must also be considered, for example, age and the geographical locations of incidence. Evidence has shown that in the U.S. for example, 95% of all the cases of antigenic bacterial infections occur in children less than 5 years of age[6]. *H. influenzae* are chiefly observed in children aged from 1 month to 6 years and it is the most common agent in the U.S. From the age 6 years, patients are most susceptible to meningococcal meningitis. Among all ages, *H. influenzae* type b, *N. meningitides*, and *S. pneumoniae* have the highest incidence[2]. The prevalence of these bacteria is probably due to poorly developed immune systems of the neonates. Several anecdotal accounts[6,7,8] support the hypothesis that viruses induce immune suppression and predispose to bacterial infections. Consequently, monocyte chemotaxis, polymorphonuclear cell functions are impaired. Other documented evidence confirms that viral infections precede and not coincide with bacterial infection[1,2,6,7,8].

Variations in virulence between the streptococcal strains that cause different types of respiratory tract infections, influenza-like illness, the common cold, and host susceptibility are influenced by extrinsic environmental, individual living conditions and personal hygiene. Several pieces of evidence are in support of these variable factors. For example, both influenza and meningococcal disease are found to show seasonal variation with peak incidence rates during the winter[9] and this seasonal variation depended on age and meningococcal phenotype, the most pronounced seasonality being in 5–14 year olds[10]. This is in consonance with our results.

In many parts of the world, for example, England, Wales, Africa, Latin America, and Asia, streptococcal diseases fluctuate with changes in the weather. For example, in England and Wales, meningococcal disease peaks in the winter months as does the incidence of both influenza-like illnesses and common cold[11,12]. Influenza often leads to bacterial complications that require treatment. It may also be confused with bacterial respiratory infections, leading to unnecessary prescription of antibiotics. The peak of influenza activity occurs during the winter. The fluctuation is relatively constant over the years with peaks in the beginning to mid December and in February–March, especially for older age groups[13].

In the sub-Saharan Africa, the incidence of influenza-like illnesses and respiratory diseases usually occur in the cold, dry, and windy harmantan season. In the U.S. on the other hand, the outbreak of influenza-like illnesses and streptococcal diseases may vary from state to state. In Houston, for example, which is the major city in Texas, streptococcal disease and other influenza-like illnesses peak in the months of January, February, March, April, and reach the highest level in the month of December. There are several anecdotal accounts of the association between the outbreak of influenza and streptococcal disease that include cerebrospinal meningitis, obstructive bronchitis, and bronchopneumonia[14,15].

Individual living conditions and personal hygiene play a very important role in the incidence of these bacterial infections within the communities. In some instances where people live in congested homes, the probability of the spread of infectious diseases is significant.

This study, although on a small scale, has in many ways identified the peak period, age, sex, and ethnic distribution and the periods at which the outbreak of these diseases are likely to occur. A most likely explanation of the factors that promote the incidence is also identified. Public health authorities should be aware of these factors that promote the infectious bacterial diseases and should be prepared to alert medical practitioners and the public, when such outbreaks occur.

## CONCLUSION

Although a positive result in these tests depended on the presence of detectable levels of antigens in the body fluids or blood, they did not serve as substitutes for culture procedures, but they were rather used for screening purposes. This was because there were a few examples of cases, where unrelated bacteria possess common antigens with other immunological test systems. Also, the possibility of cross-reactions occurring in the test could not be ruled out. Streptococci belonging to serological group b are frequently carried in man, however, infections are relatively rare and are usually mild. But the frequency of serious infections in neonates, especially meningitis and sepsis, is increasing. Some 1 to 3 cases per 100,000 births occur with a mortality of 30–60%. Perinatal infections within the first 7 days of life usually results from vaginal organisms carried by the mother. The vaginal carriage rate in women is about 30%. Although this study included a small population (2,837 patients) at risk, we believe that it confirmed the previously suggested influence of age, sex, and poverty on seasonal variation in bacterial infections. It also explained some of the probable agents that promote such bacterial infections among communities. Above all, this study will assist the public health authorities to expect, prepare, and alert the medical practitioners and the public about an increased risk of bacterial disease in any given “risk months”.

## BIOSKETCHES

**Ebere C. Anyanwu, PhD,** is a Clinical Neurophysiologist and Director of Cahers Neurosciences Research Inc, Houston. He is also an Adjunct Professor of Anatomy and Physiology at the North Harris and Montgomery Colleges in Houston and The Woodlands, respectively. Also a Researcher at the Biomarkers Research Laboratory, Department of Environmental Chemistry and Toxicology, Texas Southern University, Houston. E-mail: ebereanyanwu@msn.com

**Mohammed Morad, MD** is a specialist in family medicine, lecturer in family medicine at the National Institute of Child Health and Human Development; Division of Community Health, Ben Gurion University of the Negev and the medical director of a large area clinic in the city of Beer-Sheva. Publications include those on Bedouin health, health aspects, spiritual health and aging in persons with intellectual disability, and he is a presenter on topics like health policy and services for disadvantaged at national and international conferences. E-mail: morad62@barak-online.net

**Andrew W. Campbell**, **MD,** is a medical scientist in immunotoxicology, Medical Director and Chief Executive of the Medical Center for Immune and Toxic Disorders, Houston, TX. E-mail: md@immunotoxicology.com
